# Exploring the influence of moon phases and weather on mortality in a palliative care unit over a ten year period

**DOI:** 10.1038/s41598-025-03184-4

**Published:** 2025-05-24

**Authors:** Evelyn Mueller, Miriam Brönner, Frank Schuster, Birgitt van Oorschot, Carmen Roch

**Affiliations:** 1 Interdisciplinary Center for Palliative Medicine, Department of Radiation Oncology, University Hospital Wuerzburg, Josef-Schneider-Straße 11, 97080 Würzburg, Germany; 2 Department for Anaesthesiology and Intensive Care, Donau Isar Hospital Deggendorf, Perlasberger Str. 41, 94469 Deggendorf, Germany

**Keywords:** Mortality, Palliative care, Moon, Weather, Temperature, Medical research, Risk factors, Ecological epidemiology

## Abstract

Despite a common belief among public and sometimes medical professionals that moon phases affect mortality rates, studies do not confirm generally higher mortality rates. However, periods of extreme heat or cold have been shown to cause fluctuations in mortality, especially in vulnerable patients. This study aimed to examine whether mortality rates in a 10-bed palliative care unit were influenced by moon phases or weather conditions (temperature, barometric pressure, humidity). Data were gathered from records of the Wuerzburg University Hospital palliative care unit, the Wuerzburg weather station, and the NASA lunar calendar. Daily death rates were calculated over a 10-year period (2010–2019). Bivariate analyses and linear multivariate regression analyses were used to explore potential relationships between moon phases, weather, and mortality. Linear associations, a prerequisite for regression analysis, were ensured using Box-Tidwell tests. Over 3652 days, 3120 patients were treated, with 1508 (48.4%) deaths recorded. The average daily death rate was 0.41 (SD = 0.65), with a maximum of five deaths per day. No deaths occurred on 2411 days (66%). The average death rates were similar across the moon phases: 0.40 (SD = 0.71) for the full moon, 0.42 (SD = 0.66) for the new moon, 0.40 (SD = 0.62) for the waxing moon, and 0.43 (SD = 0.66) for the waning moon, with no significant association (ANOVA: F(3, 3648) = 0.51, *p* = 0.67). Weather variations were not significantly associated with death rates. The multivariate linear regression analysis confirmed that no combined effects, e.g., of different weather aspects, were found (F(6, 3643) = 0.41, *p* = 0.87). Our findings align with those of previous studies, which revealed no associations between the moon phases and mortality rates. Unlike general mortality trends, temperature did not significantly impact death rates in the palliative care unit, possibly because of controlled environmental factors. Limitations included a low incidence of extreme weather, which may have reduced the statistical power.

*Trial registration* This study did not involve any health care interventions for human participants and, therefore, did not require registration.

## Introduction

The beginning and end of life are often associated with various beliefs regarding the influence of natural phenomena. The assumption that the moon influences medical issues and death has been a topic throughout human history. In particular, the full moon and eclipse are associated with the expectation of higher death rates. The ancient Egyptians believed that the moon was a guide to the underworld and associated with the god Thoth, who made judgments after death. The moon has also always been a topic in medicine. Hippocrates, 2500 years ago, wrote that “no physician should be entrusted with the treatment of disease who was ignorant of the science of astronomy”^1^. Currently, approximately 40% of healthcare professionals believe in the effects of the moon on human behavior^2–4^. Staff in palliative care are no exception, with the notion of higher death rates on full moon days often being mentioned.

A large-scale study on mortality in the general population of Australia, conducted over 29 years and including all causes of death, reported no connection between mortality rates and the lunar cycle^5^. However, what about specific causes of death? A wide range of causes of death have been studied in relation to lunar phases. For example, no influence of the lunar phase has been found on admission to^6^ or mortality in intensive care^7^. The lunar phase at the time of medical procedures is not associated with complications, mortality during or long-term survival in cancer patients^8–10^, kidney transplantation^11^, or emergency surgeries^4^. Studies on cardiac events and deaths consistently find no increased risks during a full moon, despite this being a common belief; some studies have reported small protective effects during the new moon^12–15^. Neither the occurrence of nor mortality by ischemic strokes are associated with moon phases^16^. There is also no evidence supporting the influence of the moon phase on suicides^17,18^. However, the results of studies on homicides are controversial, reporting higher^19^, lower^20^ or no changes in homicide rates at full moon^21,22^. Recent studies on traffic accidents have reported slightly higher incidences during the full moon^23,24^. To the best of our knowledge, no scientific studies have explored the influence of moon phases on biochemical processes related to dying or death rates among palliative care patients.

While the influence of moon phases is largely rooted in long-standing beliefs, the effects of weather conditions on mortality are based on scientific evidence. The impacts of extreme heat and cold on mortality, particularly among high-risk groups, are well documented and increasingly discussed due to climate change^25–27^. Studies have shown that heatwaves can increase death rates, for example, by exacerbating preexisting cardiovascular diseases^28^ or causing dehydration^29^. Low temperatures increase the risk of hypothermia^29,30^, as well as heart attack and stroke^28,31^. Furthermore, cold weather facilitates the spread of respiratory viruses, such as influenza, pneumonia, and RSV, which can be fatal for vulnerable populations^32,33^. We are not aware of studies that explore whether these effects can be shown in patients in a palliative care unit.

Weather and moon phases do not directly influence each other. However, weather significantly affects the human perception and thereby possible effects of the lunar phases, e.g. clouds obscure the light of the moon and cold and wet weather reduce time spent outdoors. Against this background, we are also interested in analyzing both types of exposure together in this study.

This study aimed to examine whether mortality rates in a 10-bed palliative care unit were influenced by moon phases or weather conditions (temperature, barometric pressure, humidity).

## Methods

### Study design and setting

We conducted a retrospective analysis of patient data from a 10-bed specialist palliative care unit within the Department of Radiation Oncology at the Comprehensive Cancer Center, University Hospital Würzburg, Germany. Despite its primary affiliation with oncology, this unit also admits patients referred from other specialties, such as cardiology, neurology, and nephrology. The palliative care ward was not air-conditioned. All patients had access to a shared balcony, which was available to all patients regardless of mobility or disease status.

### Sample and inclusion criteria

Data were collected from all patients aged ≥ 18 years who died in the palliative care unit between January 1, 2010, and December 31, 2019. Data after 2019 were not included due to structural changes that included, e.g., a reduction in the number of beds and thereby limited comparability. No additional inclusion or exclusion criteria were applied.

### Data collection

Data analysis was conducted with IBM SPSS 29^34^. Patient data, including date of death, sex, age, and primary diagnosis (coded by the ICD-10), were extracted from the hospital’s clinical information system and ward records. Meteorological data for the same 10-year period, including daily mean temperature (°C), maximum and minimum temperatures (°C), mean air pressure (hPa), and relative humidity (%), were obtained from the German Weather Service online database for Wuerzburg weather station, which is located 3.6 km from the palliative care unit and has an altitude difference of about 30 m to the palliative care unit. Lunar phase data were retrieved from the NASA website and categorized into four groups: full moon, new moon, waxing moon, and waning moon.

### Datasets

Two datasets were created:

- *Patient-level dataset*: This dataset contains individual demographic and diagnostic information for a descriptive analysis of the patient sample.
- *Daily level dataset*: This dataset includes aggregated data for 3652 days, such as the total number of deaths per day, lunar phase, and meteorological data.

This comprehensive approach enabled both a description of patient characteristics and an exploration of potential associations between daily death rates and meteorological or lunar factors.

### Sample size

The sample size calculation focused on the number of days included in the dataset rather than the number of patients, as daily level data were necessary to answer the research questions. A post hoc power analysis for multiple linear regression with four predictors (moon phase, maximum temperature, barometric pressure, and humidity) and 3652 days indicated that even minimal effects (R^2^ < 0.02) could be detected with a statistical power of 0.9 at a significance level of α = 0.05^35,36^.

### Analysis

We began with a descriptive analysis of the data, including testing the normality of continuous variables (e.g., daily death rate, weather data) using the Shapiro–Wilk test. To examine bivariate associations between weather variables and death rates, we planned to use Pearson correlations or, as a robust alternative, Kendall’s tau for cases involving data that are not normally distributed and have high levels of tied ranks or outliers.

Variance analyses were conducted to determine the associations between moon phases and the daily death rate. Given the exploratory nature of the bivariate analysis, we did not adjust the significance level, and the alpha was set at 5% (two-sided). We hypothesized that combined effects of different weather and lunar phase variables might exist, even in the absence of significant bivariate correlations with the daily death rate (e.g. hot and humid vs hot and dry weather conditions; cloud-covered vs. clearly visible moon).

We calculated autocorrelations for death rates (with lags from 1 to 365 days) to assess interdependencies in daily death rates that might justify a time series analysis. Additionally, we considered various regression models: Poisson regression was ruled out due to an excessive number of days without observations. Assumptions were tested for both binary logistic regression (log-odds relationship) and multiple linear regression (linear relationship). Specifically, we checked for linearity (Box-Tidwell transformation; *p* < 0.00625, Bonferroni correction), independence of residuals (Durbin–Watson test; values close to 2 indicate independence), and homoscedasticity (Breusch–Pagan test; *p* > 0.05). If either of the latter two assumptions was violated, bootstrapping with 1,000 samples was applied to robustly estimate the regression model parameters.

We conducted both multiple linear regression (dependent variable: number of deaths per day) and binary logistic regression (dependent variable. days with vs. without death(s)) to evaluate the joint predictive power of weather data and moon phases. As multicollinearity was expected (e.g., among temperature variables), predictors were screened. Variables were excluded when multicollinearity was detected, defined as a variance inflation factor (VIF > 10) and/or a Pearson correlation (r > 0.8) between predictors. Moon phases were entered as dummy-coded, dichotomous variables for each moon phase.

### Ethics approval and consent to participate

This retrospective study utilized pseudo anonymized data. Due to the retrospective nature of the study, the Medical Ethics Committee of the Julius-Maximilians-Universität Würzburg waived the need of obtaining informed consent. This is based on the laws of Bavarian Data Protection Act (BayDSG, Article 25) and Bavarian University Hospital Act (BayUniKlinG, Article 16). The committee also confirmed that there were no ethical concerns (file number: 2025-1-ka). All relevant regulations were followed.

## Results

Over 3652 days, 3120 patients were treated in the palliative care unit, with 1508 (48.4%) deaths recorded. Table 1 summarizes the main characteristics of the patient samples (patient-level dataset).

**Table 1:** Characteristics of patient sample (n = 1508 patients).

Characteristics	Statistics	
	m (sd)	min–max
Age	69.3 (13.3)	19–100

	n	%
Gender		
Female	731	48.5
Male	777	51.5

	n	%
Primary diagnosis		
Malignant disease	1220	80.9
Nonmalignant disease	288	19.1

	m (sd)	min–max
Length of stay	8.6 (7.5)	1–69

When the number of days of death on the calendar in the daily level dataset was projected, the average daily death rate was 0.41 (SD = 0.65), with a maximum of five deaths per day. No deaths occurred on 2411 days (66%). No significant autocorrelations were found in daily death rates. The highest autocorrelation was r = 0.070 (*p* = 0.149), indicating no temporal interdependency in daily death rates. Table 2 gives details of the descriptive data regarding the daily level dataset. Neither the daily death rate nor the temperature variables, barometric pressure, or humidity were normally distributed (*p* < 0.001).

**Table 2:** Characteristics of the daily data in the 10-year period (n = 3652 days).

Characteristics	Statistics	
	m (sd)	min–max
Daily death rate	0.41 (SD = 0.65)	0–5

	n	%
Days with … patients dying		
0	2411	66.0
1	1019	27.9
2	186	5.1
3	28	0.8
4	7	0.2
5	1	0.1

	n	%
Moon phases, number of days with…		
Full moon	123	3.4
New moon	124	3.4
Waxing Moon	1696	46.4
Waning moon	1709	46.8

	m (sd)	min–max
Weather data		
Mean temperature (°C)	10.7 (7.7)	− 12.2–29.8
Maximum temperature (°C)	15.2 (6.6)	− 8.6–39.4
Minimum temperature (°C)	6.2 (6.6)	− 16.5–21.8
Barometric pressure (hPa)*	984.5 (8.0)	945–1010
Relative humidity (%)*	75.5 (13.5)	33–100

*n = 3650 due to two days with missing weather data.

### Bivariate associations

**Fig. 1:**
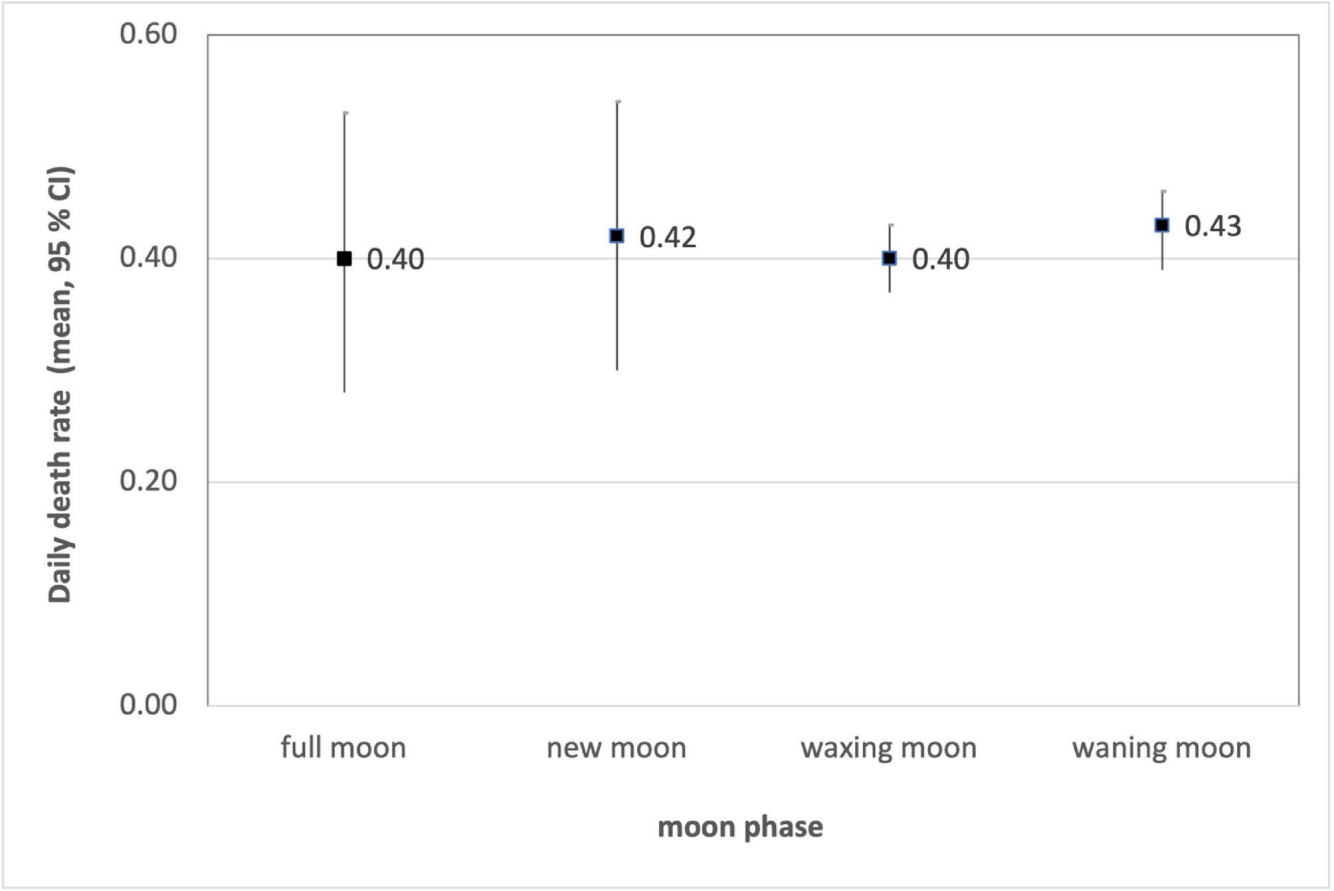
Mean values and confidence intervals of the daily death rate depending on the moon phase (ANOVA: F(3, 3648) = 0.51, *p* = 0.67).

There was no significant association between the moon phase and the daily death rate (ANOVA: F(3, 3648) = 0.51, *p* = 0.67). The mean daily death rates ranged from 0.40 on days of the waxing moon to 0.43 on days of the full moon, with the confidence intervals for the mean estimates strongly overlapping (see Fig. 1).

**Fig. 2:**
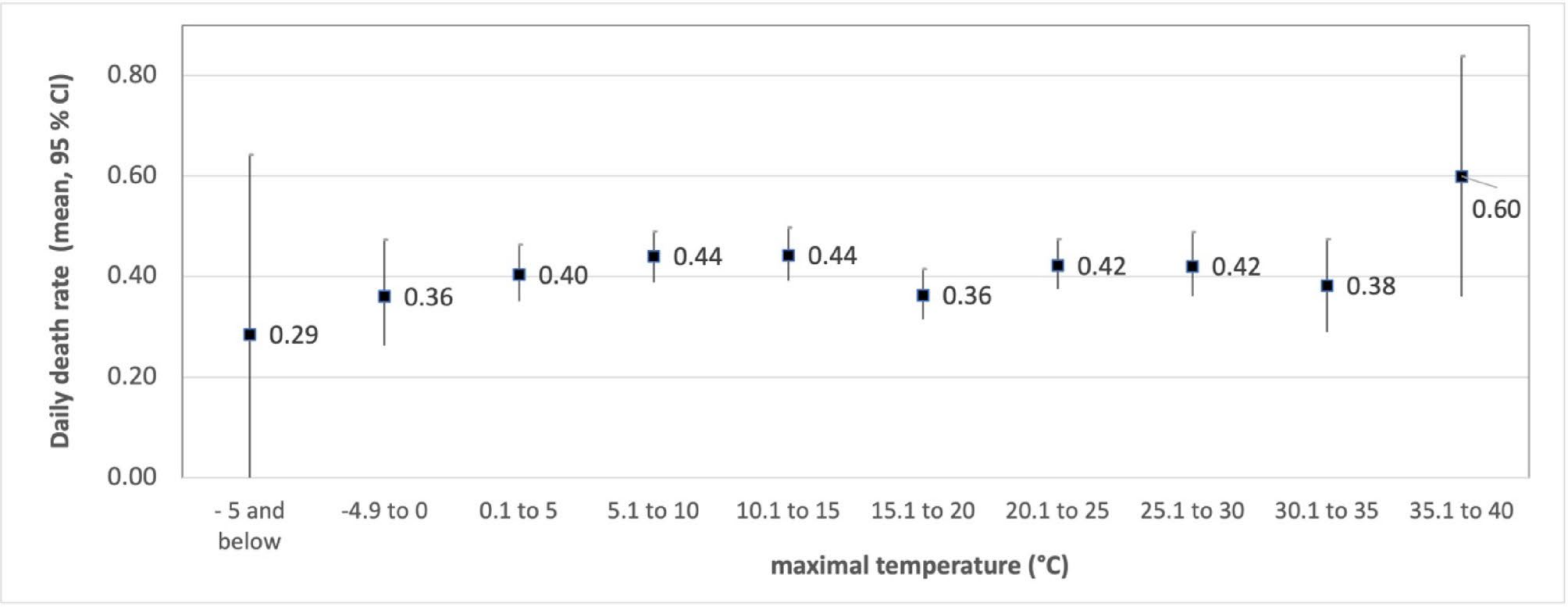
Mean values and confidence intervals of the daily death rate depending on the maximal temperature (Kendall Tau = 0.002; *p* = 0.862).

Figure 2 shows the variation in death rates as a function of the maximum temperature. While there are some differences in the mean values between categories, the overlapping 95% confidence intervals suggest that these differences are likely random rather than systematic. The categories with temperatures below -5 °C (n = 14) and 35 °C or higher (n = 25) have particularly wide confidence intervals, reflecting the imprecision in measuring death rates due to the low number of days in these temperature ranges.

Owing to deviations from normality in all-weather variables and daily death rates, Kendall’s tau was used to assess bivariate associations between weather data and daily death rates (Table 3). No significant correlations were found.

**Table 3:** Correlation coefficient Kendall tau for bivariate associations between weather data and daily death rates (number of days n = 3650–3652).

	Temperature			Barometric pressure	Relative humidity
	Max	Min	Mean		
Daily death rate	0.002	− 0.002	0.000	− 0.016	0.003

### Testing of assumptions for regression models

Due to high multicollinearity, defined as Pearson correlations exceeding 0.8 and/or variance inflation factors (VIFs) greater than 10, the mean and minimum temperatures as well as the waning moon phase were excluded as predictors. There was no evidence of nonlinear relationships among the predictors and the outcome variable (Box-Tidwell procedure). No autocorrelation of residuals was detected (Durbin–Watson statistic of 1.78). However, the assumptions of homoscedasticity (Breusch‒Pagan test *p* < 0.001) and normality of the residuals were both violated (Shapiro‒Wilk test *p* < 0.001). To address these violations, bootstrapping with 1000 samples was employed to ensure robust estimation of the linear regression model.

### Regression models

The regression models included maximum temperature, barometric pressure, relative humidity, and lunar phases, with full, new, and waxing moons entered as dichotomous variables. Both multiple linear regression analysis (R^2^ = 0.001) and binary logistic regression (R^2^ = 0.001.) indicate that the predictors collectively accounted for only 0.1% of the variance in daily death rates. The overall models were not statistically significant (linear regression: F(6, 3643) = 0.41, *p* = 0.87; binary logistic regression: Chi^2^(6) = 1.61; *p* = 0.952)), and none of the individual predictors contributed significantly to any of the models, with all *p* values exceeding 0.05.

## Discussion

Our results revealed no correlation between particular phases of the moon or weather conditions and patient death in our palliative care ward, either alone or in combination.

Our results align with those of other studies and contradict the common belief held by many people, including staff in palliative care units. How can this contrast between perception and research findings be explained? First, there are actual effects of the moon, which are speculated to be driven by its bright light, confirming beliefs in lunar influence. For example, studies in indigenous communities living without electric light have revealed synchronization between nocturnal sleep and the moon cycle^37^. This association is not found in studies conducted in industrialized communities, where artificial lighting is ever present^38^. Moon-related effects of traffic accidents^23,24^ and homicides^20,21^ have also been attributed to the brightness of the moon and its effects on human activity.

Second, humans have a natural tendency to search for patterns and rules, sometimes finding them even though they are not real (“Apophenia”)^39^. Once a person believes in such a pattern, “confirmation bias” takes over, influencing their perception to favor and even seek out information that confirms their beliefs^40^. Individual cases that support the hypothesis are overemphasized, whereas counterexamples are ignored. Through these mechanisms, cultural beliefs are perpetuated despite contradicting scientific evidence.

Interestingly, science also contributes to keeping up those beliefs: flawed methods can lead to false positive results. For example, random clusters of deaths on full moon days may skew the results of studies with small sample sizes. Furthermore, positive findings that support myths are also more likely to be published, spreading the idea that the moon influences human life and death (publication bias^41^).

With respect to the effects of weather, particularly extreme temperatures, our findings deviate from those of studies in the general population. Our hypothesis that high temperatures are associated with higher mortality rates in a palliative care unit without air conditioning was not confirmed.

Temperature regulation is highly effective in healthy humans^42,43^, but how these compensatory mechanisms change in the dying phase is not yet well understood. During the dying process, essential compensatory mechanisms are assumed to be lost due to factors such as disease-related inability to consume sufficient fluids or food, large wound areas with exudation and evaporation of body fluids, or fever^44^. While nutrition, fluids, and antibiotics are typically continued in the acute setting, they are often discontinued at the end of life in the palliative setting to avoid burdening the patient and to avoid delaying the dying process^45^.

While our patients were exposed to heat in the summer to some extent (no air conditioning), our patients also received continuous, professional care in the palliative care unit. This care included measures to reduce heat as much as possible and mitigate its impact on the body and health of patients. Care in this setting is highly individualized, attentive to patient needs, and designed for comfort. Therefore, even though palliative patients may have a limited ability to adapt to extreme temperatures, they are likely to be in an ideal environment that compensates for it.

Furthermore, extreme weather conditions were rare during the 10-year study period. For example, only 0.7% (n = 25) had a maximum temperature above 35 °C. This rarity likely reduces the statistical power to detect associations between extreme weather conditions and daily death rates, even though the overall dataset is large.

Limitations of the studies include the fact that this study was conducted at a single center, and the findings may have been influenced by the patient sample, local weather conditions, room characteristics (e.g., no air conditioning), and ward-specific strategies for managing extreme weather. As a result, the findings might not be generalizable, e.g., to countries with more extreme weather conditions.

For a completely accurate comparison of daily death rates, adjustments based on the daily occupancy of the palliative care unit would have been ideal. However, tracking daily occupancy over 10 years would have been highly impractical. The occupancy figures remain very stable within each year, showing an overall increase over time in annual occupancy from 7.4 to 9.0 out of the 10 beds. Despite this increase, at least 8 out of the 10 beds were occupied on 83% of all days over the 1.0-year period—with even less variation within each specific year.

## Conclusion

Our findings on the lunar phase align with those of previous studies, which revealed no associations between the lunar phase and mortality rates for the vast majority of potential causes of death. Unlike general mortality trends, temperature did not significantly impact death rates in the palliative care unit, despite the assumed vulnerability of seriously ill palliative patients, possibly due to controlled environmental factors and individualized care in the palliative care unit.

## Data Availability

The datasets used and/or analyzed during the current study are available from the corresponding author upon reasonable request.
